# Flow Injection Amperometric Evaluation of Trolox Equivalent Antioxidant Capacity of Chocolates with Different Cocoa Content at a Boron-Doped Diamond Electrode

**DOI:** 10.17113/ftb.59.02.21.6984

**Published:** 2021-06

**Authors:** Tahir Arbneshi, Arbër Frangu, Michaela Frühbauerová, Libor Červenka, Liridon Berisha, Kurt Kalcher, Milan Sýs

**Affiliations:** 1 Department of Chemistry, Faculty of Mathematics and Natural Sciences, University of Prishtina, Str. Mother Teresa, 10 000 Prishtina, Republic of Kosovo; 2 Department of Analytical Chemistry, Faculty of Chemical Technology, University of Pardubice, Studentská 573, 532 10 Pardubice, Czech Republic; 3 Institute of Chemistry-Analytical Chemistry, Karl Franzens University, Universitaetsplatz 1, 8010 Graz, Austria

**Keywords:** Trolox equivalent antioxidant capacity, amperometry, boron-doped diamond electrode, flow injection analysis, cocoa mass fraction in chocolate

## Abstract

**Research background:**

The objective of this paper is to introduce an instrumentally simple analytical tool for determination of cocoa solid content in chocolates. This electroanalytical method is based on amperometric oxidation of all present antioxidants in chocolates at boron-doped diamond electrode (BDDE) that is integrated in a flow injection analysis (FIA) wall-jet electrode system.

**Experimental approach:**

As part of optimisation, thirteen commonly occurring antioxidants were investigated using cyclic voltammetry at the BDDE in 0.1 mol/L phosphate buffer with different methanol (MeOH) content. Working parameters, such as MeOH volume fraction, flow rate and detection potential, were optimised. Principally, the height of the oxidation peak (current response) representing the oxidation of the sum of antioxidants (total antioxidant content; TAC) was expressed as Trolox equivalents.

**Results and conclusions:**

For analytical purpose, a linear range from 5 to 100 mg/L described by regression equation and characterised by high correlation coefficient R^2^=0.9994 was achieved. Obtained high positive correlation between the determined values of Trolox equivalent antioxidant capacity (TEAC) and cocoa mass fractions characterised by correlation coefficient of 0.9187 for eight randomly selected samples (one white, two milk, and five dark chocolates) confirmed that cocoa solids represent the main source of antioxidants (reducing agents).

**Novelty and scientific contribution:**

The research demonstrates that TEAC values could be considered as an additional marker of cocoa content in the chocolate analysis to the commonly used theobromine (authenticity of food products). The developed FIA could therefore serve as simple analytical tool in the food quality control.

## INTRODUCTION

Chocolate is a favourite food product made from cocoa beans that is consumed as sweets or beverage and to flavour or coat various confectionery and bakery products (*1*). Generally, the chocolate is divided into three main categories, namely dark, milk and white chocolate (*1*, *2*). Dark chocolate usually contains 50-90% cocoa solids, cocoa butter and sugar, whereas milk chocolate contains 10-50% cocoa solids, cocoa butter, milk in some form and sugar. White chocolate does not contain any cocoa solids and is made simply of cocoa butter, sugar and milk powder (*3*). Lower quality chocolates may also contain butter fat, vegetable oil or artificial colours or flavours. According to EU legislation (2000/36/ES), the last-mentioned type must not be labelled as chocolate (*4*). U.S. Food and Drug Administration (FDA) issued an order that semisweet chocolate must contain a minimum of 35% chocolate liquor (*5*).

In the recent past, Czech Agriculture and Food Inspection Authority revealed the sad fact that most commercially available chocolates do not have the declared content of cocoa solids in order to be classified as a regular chocolate. Moreover, the statutory minimum content of cocoa solids was missing in some chocolate drinks (*6*). These unsatisfactory reports demonstrate the urgency to develop a simple analytical method applicable in the chocolate analysis.

Theobromine (TBR) is the primary alkaloid contained in cocoa powder and chocolate. Since TBR ranges from 26 g/kg in cocoa to 140 mg/kg in cocoa butter, this alkaloid can be considered as a marker of cocoa content (*7*). Determination of fat-free cocoa solids is performed using a protocol ČSN 56 0578, based on the HPLC analysis (*8*).

In addition to TBR, dark chocolate is rich in minerals, such as potassium, iron, magnesium, copper, manganese and zinc. The cocoa in dark chocolate also contains antioxidants called flavonoids, which may provide several health benefits (*3*, *9*). Assuming that cocoa powder and cocoa butter are the only sources of antioxidants, it is possible to use the total antioxidant content (TAC) as another potential marker of cocoa content (*10*). Phenolic compounds, flavours (vanillin and ethylvanillin) and alkaloids (TBR and caffeine) present in chocolate represent reducing agents that can be electrochemically oxidised at carbon-based working electrodes (*11*-*14*).

Due to an insignificant passivation of the electrode surface, a boron-doped diamond electrode (BDDE) was integrated into wall-jet flow cell to find out whether a simple flow injection analysis (FIA) with amperometric detection could be used for evaluation of dark chocolates (*15*). A correlation (R or R^2^), known as a statistical measure describing a relationship between two variables (*16*), represented ideal tool to clarify the dependence between the declared cocoa content and TAC values in numerous dark chocolate samples.

## MATERIALS AND METHODS

### Chemicals and reagents

Analytical standards of ≥99.0% l-ascorbic acid, ≥98.0% caffeic acid, 99.0% caffeine, ≥99% *trans*-cinnamic acid, ≥98% (-)-epicatechin, 97% (±)-6-hydroxy-2,5,7,8-tetramethylchromane-2-carboxylic acid (Trolox), ≥95% chlorogenic acid, 97.5-102.5% gallic acid, ≥97.0% kaempferol, ≥98% (+)-catechin hydrate, ≥95% naringin, ≥98% sinapic acid, ≥98.0% theobromine and ≥97% vanillin were purchased from Sigma-Aldrich, Merck (Prague, Czech Republic). All voltammetric measurements were performed in their 1.0 mmol/L aqueous solutions of 0.1 mol/L phosphate buffer, pH=7.0, prepared from sodium dihydrogen phosphate dihydrate and disodium hydrogen phosphate, both obtained from Lach-Ner, Ltd. (Neratovice, Czech Republic). Due to low solubility, naringin, (+)-catechin, (-)-epicatechin and kaempferol had to be dissolved in phosphate buffer containing volume fraction of 10 and 50% methanol (MeOH). Deionized water (*ρ*=18.3 MΩ cm) obtained with a Milli-Q^®^ water purification system from Merck (Darmstadt, Germany) was used for the preparation of phosphate buffer.

### Pretreatment of boron-doped diamond electrode

A commercially purchased boron-doped diamond electrode (BDDE) with boron to carbon ratio of 1:1000 and a surface diameter of 3 mm (Windsor Scientific Ltd, Slough, UK) was used for all experiments. The BDDE surface was mechanically pretreated by carefully polishing it with a wet filter paper to eliminate the passivation layers on the electrode caused by oxidation products of polyphenols.

### Instrumentation

The electrochemical behaviour of the dominant thirteen substances with antioxidant effect present in chocolate and Trolox was studied using cycling voltammetry at BDDE which was simultaneously connected with a silver/silver chloride electrode, 3.0 mol/L KCl as salt bridge (reference electrode) from Metrohm Česká republika s.r.o. (Prague, Czech Republic) and platinum sheet (auxiliary electrode) from Elektrochemické detektory, s. r. o. (Turnov, Czech Republic) to the potentiostat/galvanostat Autolab PGSTAT101 operated *via* the Nova 1.11 software from the above-mentioned Metrohm company (*17*).

**Fig. 1:**
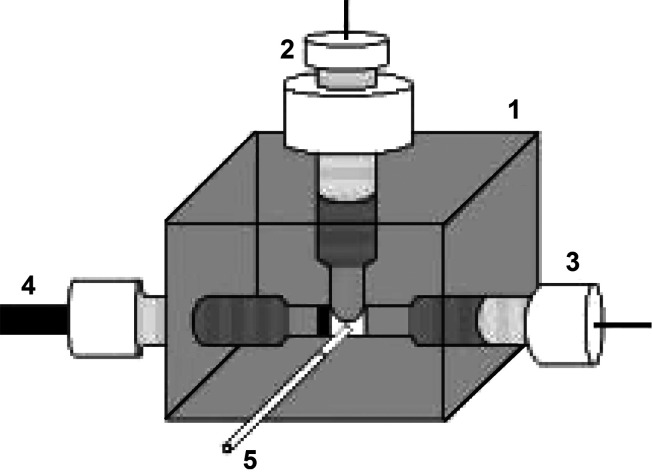
Schematic diagram of the electrochemical flow cell used in the amperometric measurements in flow injection system: 1=polyurethane resin block, 2=silver chloride reference electrode, 3=auxiliary platinum electrode, 4=boron-doped diamond electrode, and 5=polyethylene tubing

Flow injection analysis (FIA) configuration consisted of a multi-channel peristaltic pump MINIPULS 3 from Gilson (Middleton, WI, USA), Rheodyne automatic six-position dosing valve from IDEX Health & Science (Wertheim, Germany), and BDDE inserted into the cross-flow cell from Inventek Sp. z o.o. (Warsaw, Poland), as shown in Fig. 1.

### Methods

Repetitive cyclic voltammetry (five cycles) was used to determine oxidation peak potentials of the investigated antioxidant substances. Potential range was set from -0.4 to +1.6 V, initial potential of 0 V, scan rate (*ν*) 50 mV/s, and potential step (*E*_step_) 2.5 mV. Flow injection analysis with amperometric detection in the wall-jet configuration was usually performed at +1.3 V *vs* a miniature silver/silver chloride reference electrode at flow rate of 1 mL/min. The 0.1 mol/L phosphate buffer (pH=7.0) containing 30% methanol was used as flowing carrier solution. Otherwise, all necessary changes in the working conditions are listed in the legends of the corresponding figures.

### Sample preparation

Several purposefully selected chocolates of imported origin, differing in the cocoa solid content from 0 to 80%, were purchased from common stores in Prishtina, Kosovo. The extraction of potential antioxidants from the chocolate samples of 5 g containing different amounts of cacao were carried out in a total mixture of 50 mL of water (70%), acetone (29.8%) and glacial acetate acid (0.2%) using the ultrasonic bath at 30 ºC for 30 min. The acetone was evaporated in ultrasonic bath at 40 °C for 20 min. After this, the solution with chocolate was adjusted with 0.1 mol/L NaOH to pH=5 and diluted in 100-mL volumetric flask using 0.1 mol/L phosphate buffer (pH=7.0) and MeOH (*φ*=30%). The sample was then centrifuged five times at stirring speed of 1000 rpm for 4 min and filtered through a filter paper of pore size less than 1 μm. The filtrate obtained from the chocolate extracts was diluted fivefold to reduce the high content of extract-reducing agents. Sample volume of 100 µL was used for FIA analysis.

### Statistical evaluation

Analysis of chocolate extracts was always repeated five times (*N*=5) and final results were calculated and presented as error bars (confidence intervals) *x̄*±*st*_1-α_, where x̄ is the arithmetic mean, *s* the standard deviation, and *t*_1-α_ the critical value of Student's *t*-distribution for five (4 degrees of freedom) determinations (2.7764) at a significance level α=0.05 (95% probability).

## RESULTS AND DISCUSSION

### Electrochemical behaviour of substances contained in chocolate

In this work we investigated only the reducing power of the chocolate samples, *i.e.* the potential of a substance to reduce another substance either by removal of hydrogen atom or release of electrons. We did not use conventional spectrophotometric assays, which are based on monitoring the reactions between the present antioxidants and 2,2'-azino-bis(3-ethylbenzothiazoline-6-sulfonic acid (ABTS^•+^) or di(phenyl)-(2,4,6-trinitrophenyl) iminoazanium (DPPH^•^) radicals.

Since we can anodically oxidise most of the chocolate components with antioxidant activity at carbon-based working electrodes (*18*-*20*), we investigated the electrochemical behaviour of thirteen selected antioxidants using repetitive cyclic voltammetry (five cycles) at BDDE in 0.1 mol/L phosphate buffer (pH=7.0) from 0 to +1.6 V and back to -0.4 V. Due to low water solubility, phosphate buffer with MeOH (*φ*=10%) was used for electrochemical study of (+)-catechin, (-)-epicatechin and naringin, while addition of 50% MeOH was necessary for kaempferol due to its low solubility in water.

To set a constant working potential for the subsequent amperometric detection, it was important to determine the values of the peak potentials of individual antioxidants. All investigated antioxidants provided minimally one oxidation peak, where for the analytical purpose (determination of cocoa powder content in chocolate), peak potential values of the first peaks are shown in ascending order as follows: caffeic acid at +0.398 V, kaempferol at +0.483 V, chlorogenic acid at +0.505 V, sinapic acid at +0.620 V, gallic acid at +0.635 V, (+)-catechin at +0.640 V, l-ascorbic acid at +0.649 V, vanillin at +0.688 V, (-)-epicatechin at +0.744 V, naringin at +1.011 V, cinnamic acid at +1.133 V, caffeine at +1.384 V, and theobromine (TBR) at +1.404 V.

**Fig. 2:**
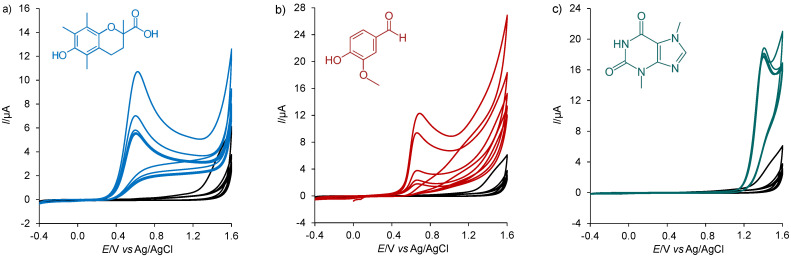
Repetitive cyclic voltammograms (5 cycles) of: a) 1 mmol/L Trolox, b) vanillin, and c) theobromine recorded on boron-doped diamond electrode in 0.1 mol/L phosphate buffer (pH=7.0) at a scan rate of 50 mV/s. Black curves (blank) indicate the cyclic voltammograms obtained for phosphate buffer only

For demonstration, repetitive cyclic voltammograms (5 cycles) of Trolox, vanillin, and TBR are shown in Fig. 2. In all cases, a decrease in the oxidation signal was observed with each subsequent cycle, indicating a slow transport of oxidation products from the BDDE surface. This phenomenon was solved when these products were flushed from the electrode surface by amperometric detection in a flow mode. From the above-mentioned peak potential values, it is clear that if a constant potential is set for amperometric detection of +0.623 V (Trolox), antioxidants having higher oxidation peak potentials will not be included in chocolate analysis. Hence, an effect of amperometric detection on total antioxidant content (TAC) values was essential for optimisation. These TAC values are usually expressed as Trolox equivalent antioxidant capacity (TEAC) (*21*). The presence of short-chain alcohols in phosphate buffer (aqueous-alcoholic mixtures) generally has no effect on peak shift. However, this assumption had to be verified for a MeOH volume fraction of 10 to 50%.

### Optimisation of flow injection analysis

The optimisation procedure included selection of optimal working parameters, such as composition of carrier solution, potential of amperometric detection, and flow rate. Due to the presence of slightly water-soluble phenolic acids, flavonoids and tannins, it was necessary to select the MeOH volume fraction in the carrier solution of0.1 mol/L phosphate buffer (pH=7.0). The whole optimisation was carried out in the dark chocolate extract with *w*(cocoa)=80%.

In general, polyphenolic compounds can be defined as weak organic acids for which it is known that their peak potentials are shifted to more positive potentials with decreasing pH values (*22*, *23*). The main reason for not performing FIA with acidic carrier solution is the necessity of amperometric detection at high positive potentials. The effect of pH in a range of pH=6-9 on current response of 1 mmol/L Trolox was investigated using cyclic voltammetry in 0.1 mol/L phosphate buffer. The obtained results indicate that the Trolox provides the maximum current response at pH=7, which was consistent with other studies that report the determination of polyphenols using FIA (*24*, *25*).

**Fig. 3:**
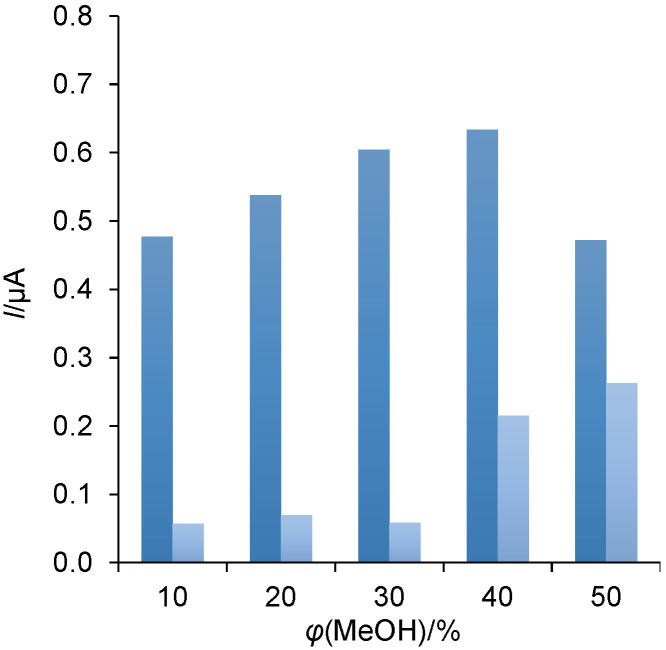
Effect of MeOH volume fraction in phosphate buffer on the current response of the extract of dark chocolate with *w*(cocoa)=80%. Results were recorded on boron-doped diamond electrode in FIA mode at a flow rate of 1 mL/min and detection potential of +1.3 V. The light blue bars indicate the baseline current responses

The optimum volume fraction of methanol in the carrier phosphate buffer solution was determined by varying *φ*(MeOH)=0-50%. For constant detection potential of +1.3 V and flow rate of 1 mL/min, the extract of dark chocolate provided an oxidation peak whose height increased with higher volume fractions of MeOH (up to 30%) in the phosphate buffer (Fig. 3) and this was taken as an optimum for further measurements.

Retaining the detection potential constant throughout the analysis is of critical significance for the application of amperometric sensing. After injection of the chocolate extract into the flowing carrier solution, an evident increase in the current response became clear for potentials greater than +0.7 V, whereas setting at higher potential values triggered only a small increase in the current response. However, a significant increase in the baseline current response was observed at detection potentials greater than +1.4 V and thus the optimal value of +1.4 V was chosen for preventive purposes.

The carrier solution flow rate was also the important FIA working parameter to be optimised as it specifies the duration of reducing agents (polyphenols) in the column where their electrochemical oxidation takes place. The flow rate of 0.2 to 1.6 mL/min for 50 mg/L Trolox was investigated at the fixed potential of +1.3 V. A sharp rise in peak height was seen up to 1 mL/min, while a constant current response was observed above that flow rate. Therefore, a flow rate of 1 mL/min was chosen as optimum.

### Analytical method validation

First, it is necessary to note that the presented contribution is not an introduction of a newly developed analytical method for TEAC determination of chocolate extracts, but an initial study to find out whether TEAC values can be used as a marker for cocoa content in chocolate samples. However, a simple validation of FIA with amperometric detection at BDDE had to take place.

Precision, defined as the level of agreement of repeated measurements, was determined as relative standard deviation (RSD) of five analyses (injections). For example, RSD values of 3.3 and 3.8% for milk chocolate (30% cocoa) and dark chocolate (50% cocoa) extracts, respectively, were calculated. If significance level of 5% (α=0.05) is taken into account, satisfactory precision can be obtained.

**Fig. 4:**
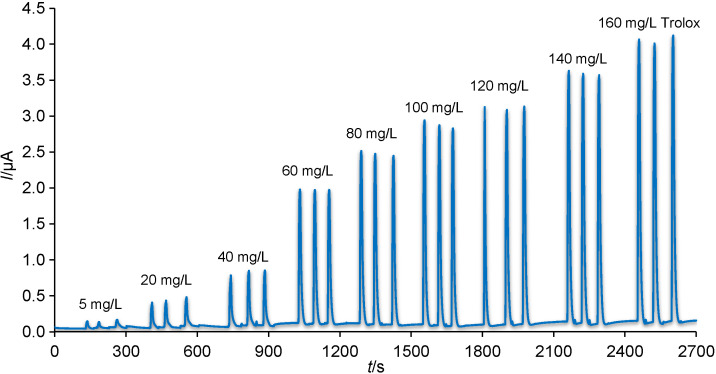
Typical amperograms of flow injection analysis recorded on boron-doped diamond electrode within calibration measurements at optimum working conditions (phosphate buffer with *φ*(MeOH)=30%, flow rate of 1 mL/min and detection potential of +1.3 V)

As shown in Fig. 4, the dependence of height of oxidation current on Trolox concentration was studied for calibration range from 5 to 160 mg/L. A calibration range from 5 to 100 mg/L Trolox was described by the following equation:

*I*=0.04859+0.0233*c* R^2^=0.9994 /1/

where 0.04859 is a slope characterising the sensitivity, 0.0233 is y-intercept, and *c* is the concentration of the standard (Trolox). This linear behaviour between Trolox concentration and peak current response can be applicable for analytical purpose. If concentrations higher than 100 to 160 mg/L Trolox are included into calculations of linear regression, the following equation will be obtained:

*I*=0.05736+0.0211*c* R^2^=0.9954 /2/

where 0.05736 is the slope, and 0.0211 is y-intercept. Due to the high value of the intercept, it was not possible to use the method of standard addition, and therefore method of calibration curve was preferred. Limit of detection (LOD) and limit of quantification (LOQ) of 1.4 and 4.6 mg/L Trolox, respectively, were calculated according to the formulae:

LOD=3*s*/*k* /3/

and

LOQ=10*s*/*k*/4/

where 3 and 10 are statistically recommended multiples of the baseline noise, *s* represents the standard deviation of five repetitive measurements of 5 mg/L Trolox and *k* is the slope of linear regression (0.0233).

### Analysis of chocolate samples

**Fig. 5:**
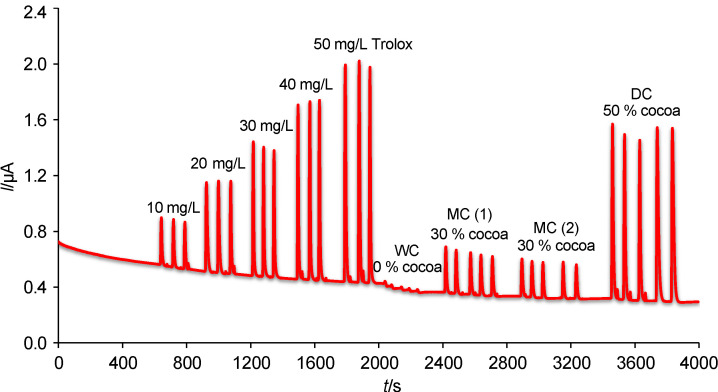
Typical record obtained during flow injection analysis of white (WC), milk (MC) and dark chocolate (DC) at the boron-doped diamond electrode

Extracts of white chocolate (0% cocoa), two samples of milk chocolate (30% cocoa), and three dark chocolates (50, 64 and 80% cocoa) were analysed using FIA at BDDE. Two milk chocolates from different manufacturers with the same cocoa mass fraction were chosen to verify the accuracy of the analysis. Fig. 5 shows that both extracts of milk chocolates provided comparable current response. In addition, a current response at the limit of detection was obtained for the extract of white chocolate which confirms that this type of chocolate cannot be considered as a rich source of antioxidants. Unlike this, the dark chocolate extract samples were diluted twice so that their current responses would not exceed the linear range.

Except for one sample of chocolate with 80% cocoa (excluded from statistical evaluation), TEAC values (mg Trolox per 100 g sample) increased with higher cocoa mass fraction. The reason why the dark chocolate extract provided the current response like chocolate samples with half the cocoa content has not been further investigated. However, it can be assumed that the manufacturer probably declared false nutritional information.

**Fig. 6:**
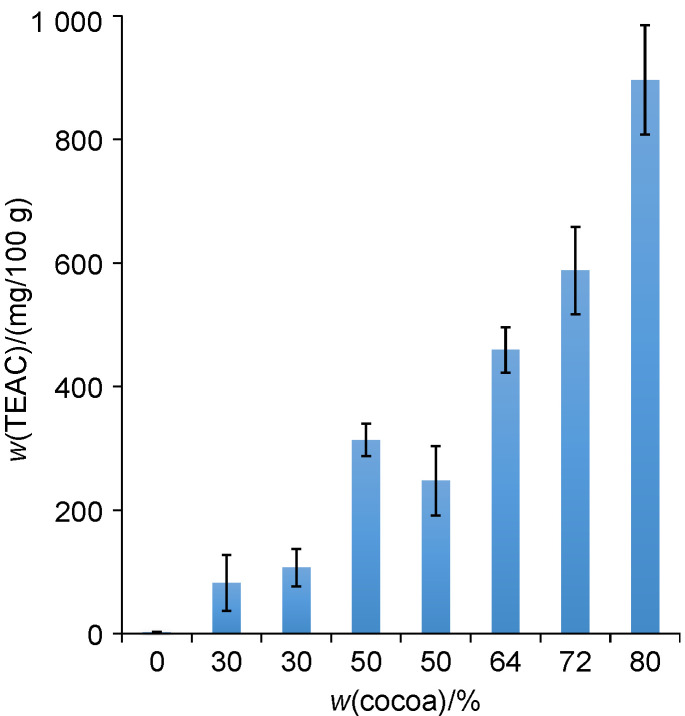
Trolox equivalent antioxidant capacity (TEAC) of white (0%), milk (30%), and dark (50-80% cocoa mass fraction) chocolates obtained using the flow injection analysis with integrated boron-doped diamond electrode

Fig. 6 shows that TAC presented as TEAC could be considered as additional marker of cocoa content in the chocolate analysis to the commonly used TBR and caffeine (*7*). Moreover, a high positive correlation between the determined TEAC values and cocoa mass fractions characterised by R=0.9187 for eight randomly selected chocolate samples is proof of that. The calculated TEAC values from FIA are in close agreement with those previously reported routine spectrophotometric assays that are usually based on the reaction of antioxidants with a colour radical (*26*, *27*).

## CONCLUSIONS

The boron-doped diamond electrode integrated in the flow injection analysis (FIA) system could represent a simple analytical tool for evaluation of chocolate quality by determining its cocoa content. This basic study represents the first step in the development of a simple analytical method for determination of cocoa content as a source of polyphenols and other potential antioxidants (reducing agents). It is expected that the analyses of more chocolate samples containing different cocoa powder mass fractions and comparisons with measured total phenolic content as Trolox equivalents will be the subjects of the upcoming investigations. The developed FIA will find application in the food quality control if the presented assumption is confirmed.
